# Evaluation of a Standard Handover Tool at a Pediatric Tertiary Care Unit in Oman

**DOI:** 10.7759/cureus.43088

**Published:** 2023-08-07

**Authors:** Hilal Al Riyami, Sharifa Al-Makhmari, Sarah Al Balushi, Saif Al Abri, Majid Al Jabri

**Affiliations:** 1 Child Health Department, Sultan Qaboos University Hospital, Muscat, OMN; 2 Child Health, Oman Medical Specialty Board, Muscat, OMN

**Keywords:** communication, patient safety, on-call handover, quality of care, i-pass

## Abstract

Background

The handover system is a great communication tool physicians use to transfer and receive patients’ care-related information. The introduction of structured handover tools has resulted in a dramatic reduction in hospital-acquired injuries. We hypothesize that the I-PASS handover tool will improve both written and verbal communication without compromising the handover duration. The current study aims to improve the quality of care and patient safety by evaluating the applicability of I-PASS handover in the Child Health Department at Sultan Qaboos University Hospital, Oman.

Results

A total of 20 trainees were enrolled in this study. After the implementation of I-PASS, 70% (14/20) of the respondents thought that the handover was well-structured, compared to 30% (6/20) prior to the implementation of I-PASS (P = .003). Due to I-PASS, about 80% of the participants could identify deteriorating patients and around 60% were confident in addressing emergencies. The I-PASS handover technique has raised participants’ satisfaction from 80% to 95%. Before I-PASS, the mean adherence rate across all 10 variables was 28.7/50 (57.4%), compared to the post-I-PASS rate of 47/50 (94%).

Conclusion

The I-PASS system is a feasible and flexible clinical handover tool. This study showed that I-PASS has improved on-call handovers and patient safety.

## Introduction

The handover system is a great communication tool physicians use to transfer and receive patients’ care-related information [1]. Handover system within the medical field has gone through significant improvements over the last few decades, to ensure patient safety [2]. The introduction of structured handover tools has resulted in a dramatic reduction in hospital morbidity and mortality [3]. It is notable that most hospital-acquired injuries are due to miscommunication, and around half of this miscommunication takes place at handover [4]. For instance, about 11% of preventable adverse events in Australia were due to miscommunication [5].

Usually, training on proper handoff tools can reduce harm to the patients and prevent the risk of missing important patient data [6]. Locally, such training does not exist for physicians. The Royal College of Physicians and Surgeons of Canada has considered handover skills (i.e. verbal and written) as an important competency measure for physicians [7].

Starmer et al. (2014) study acknowledges that miscommunication and serious medical mistakes could be overcome efficiently by using a standard tool for handover [8]. At Sultan Qaboos University Hospital (SQUH), the use of a non-standardized handoff tool has called for the introduction of I-PASS, to unify the handoff throughout the hospital. l-PASS refers to illness severity, patient information, action list, situational awareness and contingency planning, and synthesis by the receiver [7].

In the past decade, the I-PASS handover has gained popularity due to the robust evidence underpinning it and its flexibility and adaptability to clinical practice [9]. The I-PASS handover was studied in nine different USA and Canadian pediatric tertiary centers between 2011 to 2013 [9], and it was deemed to be the primary handoff tool for many pediatric departments in North America [10-14]. Looking at the Arab World, there is a lack of data regarding the use of I-PASS. Hence, it is paramount to add evidence to the entire body of knowledge by evaluating the effectiveness of the I-PASS handover in Oman.

We hypothesize that the I-PASS handover tool will improve both written and verbal communication without compromising the handover duration. The current study aims to improve the quality of care and patient safety by evaluating the applicability of the I-PASS handover in the Child Health Department at SQUH, Oman.

## Materials and methods

A prospective study was conducted in the Child Health Department of SQUH, Oman. Ethical approval was obtained from SQUH Medical Research Ethics Committee (MREC #2839). A total of twenty general foundation program (GFP) doctors, residents, and interns from two inpatient units were recruited as the study sample. In our department, a senior resident covers the call duty with the help of one junior resident and two other trainees, either a GFP or an intern. We do have a handover system but not following specific tools. We evaluated the existing departmental handover tool over the course of one week, focusing on a review of handover sheets and surveys that were filled out by all the participants.

Before the adaptation of I-PASS, a free I-PASS handover sheet from the website http://ipasshandoffstudy.com was used, and an interview with one of the study authors to clarify the process was conducted.

Lectures using PowerPoint presentations and videos were utilized to conduct I-PASS instruction sessions for all trainees of the general pediatric unit. Subsequently, the participants were instructed and supervised on the correct use of the I-PASS method. Additionally, we assessed the handover sheets after implementing the I-PASS handover, and a survey was completed by the end of the week. During this period the handover was observed by the principal investigator on a daily basis. Regular feedback was given to direct the trainee for proper handover.

Measurable variables were identified based on an online survey (yielded a total of six variables) and a review of handoff documents (yielded a total of 10 different variables to compare the two pre-I-PASS and post-I-PASS groups).

The current study included all completed handoff sheets and surveys (pre- and post-I-PASS implementation) and excluded all incomplete surveys and trainees who didn’t attend proper training on the I-PASS handover. Handoff sheets were assessed by two separate medical investigators. Later, another review was executed by one investigator for both pre and post-I-PASS implementation sheets.

Notably, to ensure survey content validity, an independent check of the survey was performed by two separate investigators. The survey findings before and after I-PASS implementation were analyzed using descriptive statistics, and the P-value was determined using the Chi-square test. A P-value of 0.05 was considered significant. The handover sheets’ information was compared, and the Chi-square test was used to identify differences between the two groups. 

## Results

Post I-PASS implementation, 70% of respondents (14 out of 20) found that the handover was well-structured, compared to 30% (6 out of 20) before I-PASS, with a significant P-value of .003.

About 80% were able to identify patients with potential deteriorations and 60% were confident to handle urgent issues after using the I-PASS. The awareness of the patient’s long and short-term plans has steadily improved after I-PASS to 80%, compared to 70% before the I-PASS.

Approximately 95% of the participants were satisfied with the I-PASS handover system, compared to 80 % before implementing the IPASS tool (Table 1). For each week before and after the I-PASS's implementation, 50 handover sheets were assessed. Comparing the pre-I-PASS handover sheets to the post-I-PASS handover sheets, a statistically significant change was seen in terms of rating the severity of the illness, the history of the present complaints, IV access, and allergies. The mean adherence rates across all 10 variables were 47/50 (94%) as opposed to 28.7/50 (57.4%) before the implementation of the I-PASS (Table 2).

**Table 1:** Pre- and post-I-PASS handover survey characteristics GFP: general foundation program I-PASS: illness severity, patient information, action list, situational awareness and contingency planning, and synthesis by the receiver

Variables	Pre (n=20)	Post (n=20)	P-value
Training level: GFP intern R1 R2 R3 R4	1 (5.0) 7 (35.0) 5 (25.0) 1 (5.0) 2 (10.0) 4 (20.0)	1 (5.0) 7 (35.0) 5 (25.0) 1 (5.0) 2 (10.0) 4 (20.0)	1.000
Organization level of handover sheet: Well Fair Not organized	6 (30.0) 14 (70.0)	14 (70.0) 4 (20.0) 2 (10.0)	0.003
Duration: Long Good	9 (45.0) 11 (55.0)	8 (40.0) 12 (60.0)	1.000
Identification of patients with potential deterioration: Yes No Not sure	13 (65.0) 1 (5.0) 6 (30.0)	16 (80.0) 1 (5.0) 3 (15.0)	0.514
Confidence to handle urgent issues after the handover: Yes Not sure	9 (45.0) 11 (55.0)	12 (60.0) 8 (40.0)	0.527
Awareness of the patient's long- and short-term plan: Yes No	14 (70.0) 6 (30.0)	16 (80.0) 4 (20.0)	0.716
Overall satisfaction: Yes No	16 (80.0) 4 (20.0)	19 (95.0) 1 (5.0)	0.342

**Table 2:** Comparison between pre- and post-I-PASS handover sheets I-PASS: illness severity, patient information, action list, situational awareness and contingency planning, and synthesis by the receiver

Variables		Pre (n=50)	Post (n=50)	P-value
Weight	No Yes	50 (100.0) -	1 (2.0) 49 (98.0)	<0.001
IV access	No Yes	37 (74.0) 13 (26.0)	3 (6.0) 47 (94.0)	<0.001
Severity	No Yes	16 (32.0) 34 (68.0)	2 (4.0) 48 (96.0)	<0.001
History of presenting complaint	o Yes	38 (76.0) 12 (24.0)	3 (6.0) 47 (94.0)	<0.001
Diagnosis	No Yes	5 (10.0) 45 (90.0)	1 (2.0) 49 (98.0)	0.204
Allergy	No Yes	49 (98.0) 1 (2.0)	9 (18.0) 41 (82.0)	<0.001
Past medical history	No Yes	4 (8.0) 46 (92.0)	4 (8.0) 46 (92.0)	1.000
To do action	No Yes	- 50 (100.0)	1 (2.0) 49 (98.0)	1.000
Medication	No Yes	4 (8.0) 46 (92.0)	1 (2.0) 49 (98.0)	0.362
Continuity plan	No Yes	10 (20.0) 40 (80.0)	5 (10.0) 45 (90.0)	0.262

## Discussion

This study has demonstrated that the current handover tool used in our unit is insufficient and could cause harm to the medical team and patients; therefore, I-PASS handover has been implemented. I-PASS is acknowledged as an essential safety tool in the health care system [4,5]. Training is the key to the proper utilization of the I-PASS handoff system. Like other medical competencies/skills, I-PASS execution requires training and monitoring [6,7]. Participants have received proper training and the handoffs were observed directly to assess adherence to the I-PASS handover tool. Overall satisfaction of 95% indicated participants’ adjustment to I-PASS and its application feasibility to other subspecialties of the unit. Moreover, 70% of the participants perceived that I-PASS was well-organized, compared to 30% and this is one of the major strengths of standardized handover [7,10].

Starmer et al. (2014) argue that when I-PASS was implemented successfully, medical errors were reduced to 23%, while preventable adverse events went down to 30% [8]. Similarly, this study found that post-I-PASS implementation, residents could identify patients with potential deterioration more quickly, and subsequently, their satisfaction level was higher.

In addition, Chang et al. described that trainees usually do not value the data they deliver at the handover time; however, proper documentation of sick patients and those who might worsen will not only reduce the rate of mistakes but will relieve the stress associated with the calls [15]. This was evident in our study as there was a significant difference in documenting the severity of the illness, history of present complaints, presence of an IV access, allergies as well as weight documentation which are all very crucial in-patient management.

Importantly, one of the main obstacles to this handover is its five components that should be followed. The last point explains the reason for having trainees skip some of the components when not being observed [16]. Blazin et al. (2020) reported a mean adherence rate of 76%-89%. Nevertheless, in our study, we have seen a higher adherence rate during the one-week observation [7]. Another drawback of this tool is its need for rehearsal which some trainees found challenging. Nonetheless, this step is very vital as it warrants the focus of the receiver during all handovers [16]. Considering the previous discussion, regular observation by expert senior residents and senior staff to guarantee appropriate guidance and evaluation was needed.

Our study has clearly demonstrated the need for standardized handover that should be part of all trainee education and evaluation. The I-PASS handover tool is the most suitable tool to be used currently in SQUH. The materials used for I-PASS have been downloaded more than 3000 times in the USA and more than 40 other countries [17]. Also, most of North American pediatric teaching units have implemented the I-PASS handover tool for quite a long time [17]. Therefore, all other units in SQUH are encouraged to adopt this handover model after appropriate training sessions. It is recommended to add the I-PASS tool to the patient electronic care system as that will ensure the continuity of such practice in a suitable format and will help clinicians access patient records more easily, prioritize tasks, and save more time.

Limitation

The study has several drawbacks. It was limited by the small sample size and restricted to just medical wards. Although we were successful in sustaining ongoing compliance and positive staff attitude toward I-PASS, a failure in establishing a connection between handoff gains and rates of adverse impacts could be noted.

## Conclusions

I-PASS is a handover tool that may be utilized in a variety of clinical situations since it is feasible, adaptable, and flexible. The current study has demonstrated that the I-PASS module has improved the caliber of the on-call handovers and made a significant contribution to patient safety and quality of care. Physicians found I-PASS as being more convenient to use and helpful in identifying and properly managing sick patients.
